# Bronchoscopic management of peripheral pulmonary lesions: robotic approach paves the way to the future

**DOI:** 10.1186/s12890-019-0927-2

**Published:** 2019-08-30

**Authors:** Michele Mondoni, Giovanni Sotgiu

**Affiliations:** 1 Respiratory Unit, ASST Santi Paolo e Carlo, Department of Health Sciences, San Paolo Hospital, Università degli Studi di Milano, Milan, Italy; 2 Clinical Epidemiology and Medical Statistics Unit, Department of Medical, Surgical and Experimental Medicine, University of Sassari, Sassari, Italy

**Keywords:** Robotic bronchoscopy, Lung cancer, Pulmonary nodules, Transbronchial biopsy

## **Editorial**

Detection and diagnosis of peripheral pulmonary lesions (PPLs), mainly those whose long axis is sized < 3 cm (i.e. nodules), challenges specialists in oncology, radiology, thoracic surgery and respiratory medicine. Their detection has increased during the last three decades owing to the widespread and frequent prescription of computed tomography (CT) [1, 2]. The early PPL diagnosis through low-dose CT was found to be associated with a 20% reduction of lung cancer specific mortality in the National Lung Screening Trial; nodules were peripheral and benign in the majority of the patients with lung nodular lesions [3]. The implementation of national screening programs will increase their notification rate (which will be closer to the real incidence rate), and, simultaneously, issues associated to their clinical management [4]. Differential diagnosis should be prioritized to exclude malignant lesions, as well as collection of sufficient tissue for molecular analysis to tailor therapies based on molecular patterns [2, 5].

A part from those nodules which deserve only radiological surveillance or which should be surgically resected, solid lesions > 8 mm should undergo non-surgical biopsies as recommended by international Guidelines, with few exceptions (e.g., in patients with low/moderate probability of malignancy, if a benign diagnosis is suspected, when clinical pretest probability and imaging findings are discordant, and when patients requires the proof of malignancy before surgical resection) [2].

Endoscopic and transthoracic approaches are available, but their prescription depends on variable covariates: lesions size, anatomical location, relation to a patent airway (i.e., CT bronchus sign), technological advances (e.g., endoscopic guidance methods), risk of complications, which could be associated with patients’ comorbidities [1, 2, 6].

Transthoracic techniques, which are mostly CT-guided, have showed the best diagnostic accuracy (≥90%) with a highest rate of adverse events (e.g., pneumothorax in > 25% of the cases) in old smokers with emphysema and with central pulmonary lesions [6, 7].

On the contrary, a lower rate of adverse events was found in patients undergoing bronchoscopy (e.g., pneumothorax in 2–5% of the cases), particularly in those with emphysema [6, 8–10]. Furthermore, endoscopic examination can investigate upper and central airways, ruling out synchronous malignancies, and favoring the collection of samples of mediastinal adenopathies [1, 2, 6, 11–13].

Bronchoscopy has showed high accuracy when prescribed for endobronchial lesions and for mediastinal staging of non-small cell lung cancer (NSCLC) [1, 6, 13].

Undeniably, ultrasound guidance and the availability of the esophageal route approach (i.e., endoscopic ultrasound with bronchoscope fine needle aspiration, EUS-B-FNA) made bronchoscopy the key procedure in mediastinal staging of NSCLC [13, 14].

PPLs investigated by fluoroscopy-guided endoscopic techniques are poorly diagnosed (sensitivity: 34–63%), particularly when the size is < 2 cm [6]. The rapid introduction of technologically advanced bronchoscopic modalities has been requested by their diagnostic effectiveness and by the healthcare need of a safe collection of clinical samples [1, 6, 8–10]. Electromagnetic navigation (EMN), radial probes endobronchial ultrasound (R-EBUS), cone beam CT, virtual bronchoscopy and ultrathin instruments, showed higher diagnostic yield (67.1–73%) in comparison with the conventional technique [1, 6, 8–10]. However, they are less accurate than CT-guided techniques, more expensive and require the recruitment of trained and skilled healthcare workers. [1, 15].

The majority of the scientific evidence can be retrieved from studies whose design is observational; only a few compared sampling techniques with a randomized controlled approach, and, then, providing low quality evidence for strong recommendations [16, 17]. Moreover, health technology assessment has not been carried out to explore economic, financial, ethical, and organizational challenges.

Combination of different guidance methods (i.e., multimodality bronchoscopy), proved an unexpected suboptimal diagnostic yield (47.1–88%) [16–19]. Newer promising techniques, such as trans-parenchymal nodule access, could detect PPLs regardless of CT bronchus sign, which is a strong predictor of success for every endoscopic technique [20, 21].

Despite the sequential introduction of several navigational modalities in the market, endoscopic sampling tools (i.e., forceps biopsy, transbronchial needle aspiration -TBNA-, brushing, etc.) did not show any changes in the last three decades [6]. However, the amount of required malignant tissue have increased for both histopathological and molecular diagnosis [5]. Few studies showed the added value of forceps biopsy and TBNA [1, 6, 16, 17, 22, 23]. Surprisingly, TBNA is an underused and underestimated technique, although it significantly helps increase the diagnostic sensitivity [17, 22, 23]. Finally, ROSE, which can allow to get high-quality specimens, cannot be performed in numerous centers [17, 22].

Few data are available on the accuracy of cryoprobes for PPLs. It can provide larger samples with a more preserved architecture, improving the quality of the specimen retrieved for the molecular diagnosis [24].

Navigational modalities should be improved for diagnostic and therapeutic purposes [21, 25].

Pre-operative placement of markers for assisting surgical resection or for guiding radiotherapy in inoperable patients represents the current clinical applications [8, 25]. Other experimental bronchoscopic therapeutic options (i.e., transbronchial brachytherapy, endoscopic radiofrequency/microwave ablation, photodynamic therapy) might show an improved safety profile if compared with percutaneous techniques [21, 25].

On this basis the best management approach would include diagnosis, staging, and therapy in the same endoscopic session, provided optimal navigation to the target lesion and confirmation of malignancy by ROSE [25].

In a recent issue of the Journal, an article described the robotic bronchoscopy, a new technological approach for the diagnosis of PPLs [26, 27]. Robotic-assisted technology, which was implemented and scaled-up more than 20 years ago, was a major breakthrough in many surgical and endoscopic procedures [28, 29].

The Monarch™ system consists of a robotically propelled outer sheath with an inner telescoping endoscope, both of which with 4-way steering control. The system relies on electromagnetic navigation for guidance with an external EM field generator. The physician uses a small hand-held controller to guide the robotic scope toward the targeted lesion. The endoscope has continuous optical capabilities, a separate suction channel, and a working channel sized 2.1 mm [21, 26, 27].

In comparison with other navigational modalities (i.e., endobronchial ultrasounds, EMN, cone beam CT) which are coupled to white light flexible instruments, robotic endoscope are specifically designed to improve access to the periphery of the lung and to sample PPLs. Preliminary conventional flexible bronchoscopy is, therefore, always necessary to explore central airways to rule out possible synchronous lesions and remove bronchial secretions.

Improved access and direct vision of peripheral airways may be a key advantage of this technique over other guidance modalities coupled with conventional bronchoscopes. Notably, this added value is not associated with a small diameter of the instrument, but to an improved structural support provided by the outer sheat, the telescoping capability, and the 4-way steering control of both outer and inner endoscope, allowing to enter airways with an acute angulation. Application of a positive end-expiratory pressure (PEEP) may induce a further advancement of the instrument in the bronchial tree [26].

Robotic bronchoscopy shows the ability to hold the endoscope in a locked curved position, favouring the placement of biopsy tools on target without straightening during sampling [26]. This technical feature, is crucial for improving diagnostic accuracy and for therapeutic purposes, based on the use of flexible ablation probes in inoperable patients [21, 24].

During the initial navigation, the outer sheat of the bronchoscope is wedged in a segmental/subsegmental airway before advancing the inner part. Beside stability improvement, it may reduce the risk of complications. Indeed, outer sheat may act as an occlusion balloon, protecting proximal airways from biopsy-related bleeding [26]. Furthermore, precise control of the bronchoscopic movements and direct visualization of small airways may enhance the safety; direct optical control of the sampling tools outside the scope and a more precise sample of the target lesion could avoid airway and parenchymal damage.

When a small airway cannot be visualized, disconnection of the proximal valve may favour pressure equilibration between the atmosphere and the airway (with or without inflating air), with the final improvement of the visualization, avoiding the injection of saline solution, which can create smear artefacts. Of note, saline alveolar filling may be associated with false positive ultrasounds and cone beam CT images, when coupled with robotic endoscopy [26].

A working channel sized 2.1 mm allows the adoption of several biopsy tools of conventional size and R-EBUS probes. Ultrasounds can support EMN guidance provided by the platform, allowing a real-time target confirmation and a precise identification of the airway/lesion relationship. Needles, forceps biopsy, and ROSE can be used to maximize the diagnostic yield [26]. Future studies may elucidate if a 1.9 mm cryoprobe might further increase accuracy.

Finally, as previously suggested, needle aspiration and artery sign (i.e., vessel leading to the lesion on the CT scans) guidance may partially help in the absence of a clear CT-bronchus sign [22, 26, 30].

A multicenter study (ClinicalTrials.gov Identifier: NCT03727425) could prove the above-mentioned potential advantages on improved navigational ability, diagnostic yield, and safety profile.

Moreover, randomized controlled trials are needed to better compare robotic bronchoscopy and other guidance methods.

Cost-effectiveness studies will be needed to assess the suitability of this advanced and expensive approach, evaluating the relationship between direct and indirect cost and clinical outcomes [26].

This new landscape in the diagnosis of PPLs could set the future bronchoscopic management designing a modern robotic perspective.

## Data Availability

Not applicable.

## Abbreviations

ACCP: American College of Chest Physicians
CT: computed tomography
EMN: electromagnetic navigation
EUS-B-FNA: endoscopic ultrasound with bronchoscope needle aspiration
NSCLC: Non-small cell lung cancer
PEEP: positive end-expiratory pressure
PPLs: peripheral pulmonary lesions
R-EBUS: radial probes endobronchial ultrasound
ROSE: rapid on-site evaluation
TBNA: transbronchial needle aspiration

## References

[1] Shepherd RW (2016). Bronchoscopic pursuit of the peripheral pulmonary lesion: navigational bronchoscopy, radial endobronchial ultrasound, and ultrathin bronchoscopy. Curr Opin Pulm Med.

[2] Gould MK, Donington J, Lynch WR, Mazzone PJ, Midthun DE, Naidich DP (2013). Evaluation of individuals with pulmonary nodules: when is it lung cancer? Diagnosis and management of lung cancer, 3rd ed: American College of Chest Physicians evidence-based clinical practice guidelines. Chest.

[3] Team NLSTR, Aberle DR, Adams AM, Berg CD, Black WC, Clapp JD (2011). Reduced lung-cancer mortality with low-dose computed tomographic screening. N Engl J Med.

[4] Oudkerk M, Devaraj A, Vliegenthart R, Henzler T, Prosch H, Heussel CP (2017). European position statement on lung cancer screening. Lancet Oncol.

[5] Planchard D, Popat S, Kerr K, Novello S, Smit EF, Faivre-Finn C (2018). Metastatic non-small cell lung cancer: ESMO Clinical Practice Guidelines for diagnosis, treatment and follow-up. Ann Oncol.

[6] Rivera MP, Mehta AC, Wahidi MM (2013). Establishing the diagnosis of lung cancer: Diagnosis and management of lung cancer, 3rd ed: American College of Chest Physicians evidence-based clinical practice guidelines. Chest.

[7] Heerink WJ, de Bock GH, de Jonge GJ, Groen HJ, Vliegenthart R, Oudkerk M (2017). Complication rates of CT-guided transthoracic lung biopsy: meta-analysis. Eur Radiol.

[8] Folch EE, Pritchett MA, Nead MA, Bowling MR, Murgu SD, Krimsky WS (2019). Electromagnetic Navigation Bronchoscopy for Peripheral Pulmonary Lesions: One-Year Results of the Prospective, Multicenter NAVIGATE Study. J Thorac Oncol.

[9] Ali MS, Trick W, Mba BI, Mohananey D, Sethi J, Musani AI (2017). Radial endobronchial ultrasound for the diagnosis of peripheral pulmonary lesions: A systematic review and meta-analysis. Respirology.

[10] Asano F, Shinagawa N, Ishida T, Shindoh J, Anzai M, Tsuzuku A (2013). Virtual bronchoscopic navigation combined with ultrathin bronchoscopy. A randomized clinical trial. Am J Respir Crit Care Med.

[11] Mondoni M, Carlucci P, Cipolla G, Fois A, Gasparini S, Marani S (2018). Bronchoscopy in patients with hemoptysis and negative imaging tests. Chest.

[12] Gasparini S, Ferretti M, Secchi EB, Baldelli S, Zuccatosta L, Gusella P (1995). Integration of transbronchial and percutaneous approach in the diagnosis of peripheral pulmonary nodules or masses. Experience with 1,027 consecutive cases. Chest.

[13] Vilmann P, Clementsen PF, Colella S, Siemsen M, De Leyn P, Dumonceau JM (2015). Combined endobronchial and oesophageal endosonography for the diagnosis and staging of lung cancer. European Society of Gastrointestinal Endoscopy (ESGE) Guideline, in cooperation with the European Respiratory Society (ERS) and the European Society of Thoracic Surgeons (ESTS). Eur Respir J.

[14] Mondoni M, D'Adda A, Terraneo S, Carlucci P, Radovanovic D, DI Marco F (2015). Choose the best route: ultrasound-guided transbronchial and transesophageal needle aspiration with echobronchoscope in the diagnosis of mediastinal and pulmonary lesions. Minerva Med.

[15] Pritchett MA, Schampaert S, de Groot JAH, Schirmer CC, van der Bom I (2018). Cone-Beam CT With Augmented Fluoroscopy Combined With Electromagnetic Navigation Bronchoscopy for Biopsy of Pulmonary Nodules. J Bronchology Interv Pulmonol.

[16] Eberhardt R, Anantham D, Ernst A, Feller-Kopman D, Herth F (2007). Multimodality bronchoscopic diagnosis of peripheral lung lesions: a randomized controlled trial. Am J Respir Crit Care Med.

[17] Oki M, Saka H, Ando M, Asano F, Kurimoto N, Morita K (2015). Ultrathin Bronchoscopy with Multimodal Devices for Peripheral Pulmonary Lesions. A Randomized Trial. Am J Respir Crit Care Med.

[18] Ost DE, Ernst A, Lei X, Kovitz KL, Benzaquen S, Diaz-Mendoza J (2016). Diagnostic Yield and Complications of Bronchoscopy for Peripheral Lung Lesions. Results of the AQuIRE Registry. Am J Respir Crit Care Med.

[19] Casal RF, Sarkiss M, Jones AK, Stewart J, Tam A, Grosu HB (2018). Cone beam computed tomography-guided thin/ultrathin bronchoscopy for diagnosis of peripheral lung nodules: a prospective pilot study. J Thorac Dis.

[20] Herth FJ, Eberhardt R, Sterman D, Silvestri GA, Hoffmann H, Shah PL (2015). Bronchoscopic transparenchymal nodule access (BTPNA): first in human trial of a novel procedure for sampling solitary pulmonary nodules. Thorax.

[21] Krimsky WS, Pritchett MA, Lau KKW (2018). Towards an optimization of bronchoscopic approaches to the diagnosis and treatment of the pulmonary nodules: a review. J Thorac Dis.

[22] Mondoni M, Sotgiu G, Bonifazi M, Dore S, Parazzini EM, Carlucci P (2016). Transbronchial needle aspiration in peripheral pulmonary lesions: a systematic review and meta-analysis. Eur Respir J.

[23] Chao TY, Chien MT, Lie CH, Chung YH, Wang JL, Lin MC (2009). Endobronchial ultrasonography-guided transbronchial needle aspiration increases the diagnostic yield of peripheral pulmonary lesions: a randomized trial. Chest.

[24] Herath S, Yap E (2017). Novel hybrid cryo-radial method: an emerging alternative to CT-guided biopsy in suspected lung cancer. A prospective case series and description of technique. Respirol Case Rep.

[25] Harris K, Puchalski J, Sterman D (2017). Recent Advances in Bronchoscopic Treatment of Peripheral Lung Cancers. Chest.

[26] Murgu SD (2019). Robotic assisted-bronchoscopy: technical tips and lessons learned from the initial experience with sampling peripheral lung lesions. BMC Pulm Med.

[27] Rojas-Solano JR, Ugalde-Gamboa L, Machuzak M (2018). Robotic Bronchoscopy for Diagnosis of Suspected Lung Cancer: A Feasibility Study. J Bronchology Interv Pulmonol.

[28] Peters BS, Armijo PR, Krause C, Choudhury SA, Oleynikov D (2018). Review of emerging surgical robotic technology. Surg Endosc.

[29] Eswara JR, Ko DS (2019). Minimally invasive techniques in urology. Surg Oncol Clin N Am.

[30] Shinagawa N, Yamazaki K, Onodera Y, Asahina H, Kikuchi E, Asano F (2007). Factors related to diagnostic sensitivity using an ultrathin bronchoscope under CT guidance. Chest.

